# Effect of Flash Light Sintering on Silver Nanowire Electrode Networks

**DOI:** 10.3390/ma13020404

**Published:** 2020-01-15

**Authors:** Dae-Jin Yang, Seyun Kim, Hiesang Sohn, Kyoung-Seok Moon, Woo Hyeong Sim, Hyung Mo Jeong, Weon Ho Shin

**Affiliations:** 1 Inorganic Material Lab, Samsung Advanced Institute of Technology, 130 Samsung-ro, Yeongtong-gu, Suwon-si, Gyeonggi-do 16678, Korea; idjyang@gmail.com (D.-J.Y.); seyuni.kim@samsung.com (S.K.); 2 Department of Chemical Engineering, Kwangwoon University, 20 Kwangwoon-Ro, Nowon-Gu, Seoul 01897, Korea; hsohn@kw.ac.kr; 3 School of Materials Science and Engineering, Gyeongsang National University, Jinju 52828, Korea; ksky.moon@gnu.ac.kr; 4 Department of Materials Science & Engineering, Kangwon National University, 1 Gangwondaehak-gil, Chuncheon 24341, Korea; 201210783@kangwon.ac.kr; 5 School of Mechanical Engineering, Sungkyunkwan University, 2066 Seobu-ro, Jangan-gu, Suwon 16419, Korea; 6 Department of Electronic Materials Engineering, Kwangwoon University, 20 Kwangwoon-Ro, Nowon-Gu, Seoul 01897, Korea

**Keywords:** silver nanowires, flash light sintering, junction structure, nanowire welding

## Abstract

We investigated the flash light sintering process to effectively reduce electrical resistance in silver nanowire networks. The optimum condition of the flash light sintering process reduces the electrical resistance by ~20%, while the effect of the conventional thermal annealing processes is rather limited for silver nanowire networks. After flash light sintering, the morphology of the junction between the silver nanowires changes to a mixed-phase structure of the two individual nanowires. This facile and fast process for silver nanowire welding could be highly advantageous to the mass production of silver nanowire networks.

## 1. Introduction

Transparent conductive electrodes (TCE) are considered one of the most important technologies for developing next generation electronic devices including liquid crystal displays, light emitting diodes, touch screens, and photovoltaic cells [1,2,3,4]. Research in the field of TCE is dedicated to the development of state-of-the-art technology that can combine high electrical conductivity with comparable optical transmittance in large-area devices [5]. Additionally, researchers have focused on introducing the flexibility of TCE components to the manufacturing of foldable/rollable electronic devices. So far, the indium tin oxide (ITO) is the most widely used material in TCE because of its high electrical conductivity and transmittance properties [6]. Much effort has been devoted to realizing flexible ITO by reducing the thickness of the conducting layer. However, the intrinsic brittleness of the oxide materials cannot withstand severe deformation, such as the 1 mm-radius bending stress necessary for foldable devices [7].

Metal nanowires (NWs), which are one-dimensional metal structures with high aspect ratio, have been proposed as the ideal material for flexible TCE due to their high electrical conductivity with low surface coverage [8,9,10,11,12,13]. Networks of NWs are highly advantageous to the industry because they can be used to produce large-area films by implementing simple coating techniques (e.g., slot-die coating, which is a common method in film-producing). Much research has been conducted to overcome the trade-off relation of electrical conductivity/transmittance in NW networks [14]. It has been demonstrated that longer metallic NWs could lead to positive effects on the electrical conductivity-transmittance relation [15]. The lengths of as-synthesized NWs are typically around several micrometres. Furthermore, silver (Ag) is considered to be the most promising material with respect to realising the feasibility of NW networks [16,17,18]. Devices containing Ag NWs have attracted much interest owing to their low-dimensional structure, which can lead to enhanced transparency and electric conductivity for applications in transparent conducting electrodes [19]. However, the insulating ligands used for Ag NW synthesis are typically made of polyvinylpyrrolidone (PVP), and the solution dispersion process can reduce electrical conductivity because of the large contact resistance between NWs [20]. Therefore, an additional fabrication process is required to reduce junction resistance between Ag NWs to optimize network conductivity.

Many methods for welding NWs have been reported, such as mechanical pressing, Joule heating, thermal sintering, electron beam-induced welding, plasmonic welding, soldering, and focused ion-beam welding [21,22,23,24,25,26]. Among them, thermal sintering, e.g., in ovens and furnaces, has been predominantly used for the sintering of NWs because it is a facile and cost-effective process. However, thermal sintering has serious drawbacks, i.e., low heating and cooling rates and long processing times. Various reports from experimental and theoretical approaches have showed that nanowire structures can easily change into nanoparticles at elevated temperatures at which the Ag atoms move easily by diffusion; a phenomenon known as Rayleigh instability [27,28,29]. To protect NW-welding from over-diffusion and breaking, precise control of the applied energy is necessary. The process of optical heating is considered suitable for welding because of its short irradiation time. While optical heating has several advantages over thermal processes, the morphological transformation of nanowires under light irradiation has been scarcely investigated [30,31]. In this study, we attempted to construct Ag NW networks via the flash light sintering process, which can selectively sinter the junction of Ag NW networks to effectively reduce electrical conductivity. Additionally, we performed a detailed analysis on the junction structure after flash light sintering to understand the mechanism behind Ag NW welding. The flash light sintering process could be an easy and cost-effective way to integrate Ag NWs into TCE devices.

## 2. Materials and Methods

Ag NWs were synthesized using a modified polyol process, as previously reported. First, 6 g of PVP was slowly added to 70 mL of ethylene glycol (EG) and completely dissolved at 60 °C. After a clear solution was formed, 2 g of AgNO_3_ was added and dissolved within 15 min. Then, an FeCl_3_ solution (0.05 M, 100 μL) was added into the mixture solution at room temperature, and the solution was placed in a pre-heated reactor to grow Ag NWs at 150 °C for 60 min. Finally, the suspension was cooled to 60 °C, and acetone and ethanol were used to wash the precipitate three times by centrifugation at 5000 rpm for 10 min.

Ag NWs were re-dispersed in ethanol and coated on an SiO_2_/Si substrate using the air spray method. The size of SiO_2_/Si substrate is 3 cm × 1 cm and the size of Ag NWs network is around 1 cm × 0.8 cm. Air pressure and needle-to-substrate distance were critical parameters for the spray coating process to ensure a uniform Ag NW network. To measure the electrical resistance, we made the electrodes on end of the prepared Ag NWs network by Ag paste. Consequently, the Ag NW networks were treated with a white light flash from a xenon lamp (ILC technology, L6755, Sunnyvale, CA, USA) comprising a wavelength range of 230–1600 nm and using a variable irradiation time. The pulse duration ranged from 1 to 5 ms, and quartz was used as a light guide. The size of spot was more than 1 cm × 1 cm, which could cover the whole Ag NW networks. The flash light was also applied on the Ag electrodes, but there was no change for Ag electrodes.

The electrical resistance of the Ag NW networks was measured using a conventional multimeter. The microstructure was investigated using scanning electron microscopy (SEM) (JSM-7600F, JEOL, Tokyo, Japan) and transmission electron microscopy (TEM) (JEM-2100F, JEOL, Tokyo, Japan).

## 3. Results and Discussion

**Figure 1:**
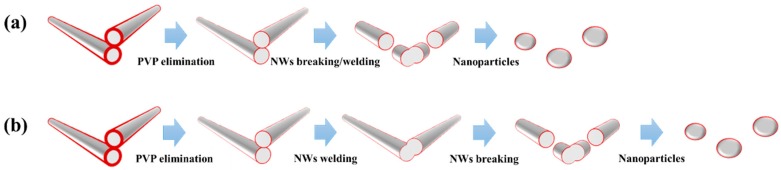
Schematic diagram of the morphology transformation of Ag nanowire (NW) under (**a**) thermal heating or high flash light sintering; (**b**) optimal condition of flash light sintering.

The sintering process for the Ag NW network is schematically illustrated in Figure 1. When the external energy, e.g., thermal heating or optical irradiation, is applied to the nanowire junction, a mass diffusion causes the morphology to change. At the first stage, the PVP layers between Ag NW junctions are eliminated owing to the low melting temperature of the material. When two nanowires come into contact, junction welding starts to minimize its surface energy. As further external energy is applied, the Ag NWs begin to break and transform to completely fragmented particles. However, typical annealing process can give the Ag NWs welding and breaking together (shown on Figure 1a) due to its high applied energy; thus, the heat treatment duration is expected to be a critical factor for determining the Ag NW weld quality and enhancing the electrical conductivity of NW networks. The short process time of flash light sintering potentially makes it a superior process for providing sufficient external energy when constructing Ag NW networks (Figure 1b).

**Figure 2:**
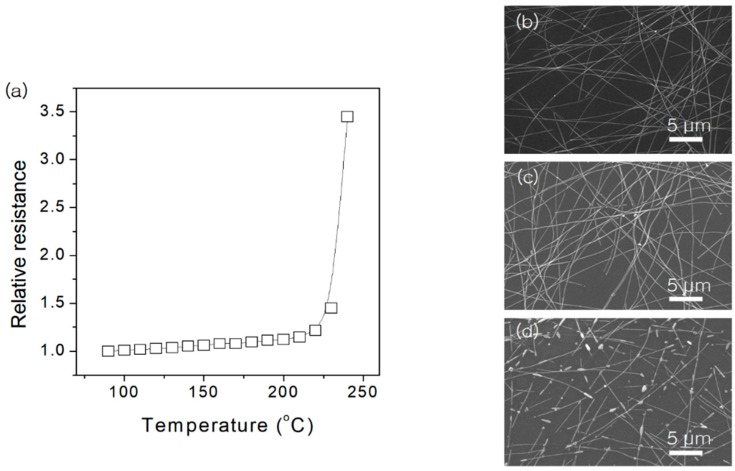
(**a**) Variation of the electrical resistance of Ag NW network during thermal annealing in air running from room temperature to 240 °C with a heating ramp rate of 5 °C/min. Morphologies of the Ag NWs after the 10-min heat treatment at (**b**) 130 °C, (**c**) 170 °C, and (**d**) 200 °C.

Figure 2a presents the change in electrical resistance of Ag NW networks during thermal heating at a 5 °C/min ramp rate at a temperature up to 240 °C. The relative resistance, R/R_0_, increases monotonically at first and abruptly when the temperature reaches 200 °C. To investigate the morphology changes in terms of the applied temperature, Ag NWs deposited on SiO_2_/Si substrates were annealed isothermally at 130, 170, and 200 °C for 10 min. SEM images of the thermal heating process at the selected temperature are displayed in Figure 2. Ag NW networks annealed at 130 and 170 °C did not show any morphological change, as shown in Figure 2a–d. However, after annealing at 200 °C, the majority of the wires broke into isolated short wires or particles, leading to a significant enhancement of the electrical resistance (Figure 2a).

**Figure 3:**
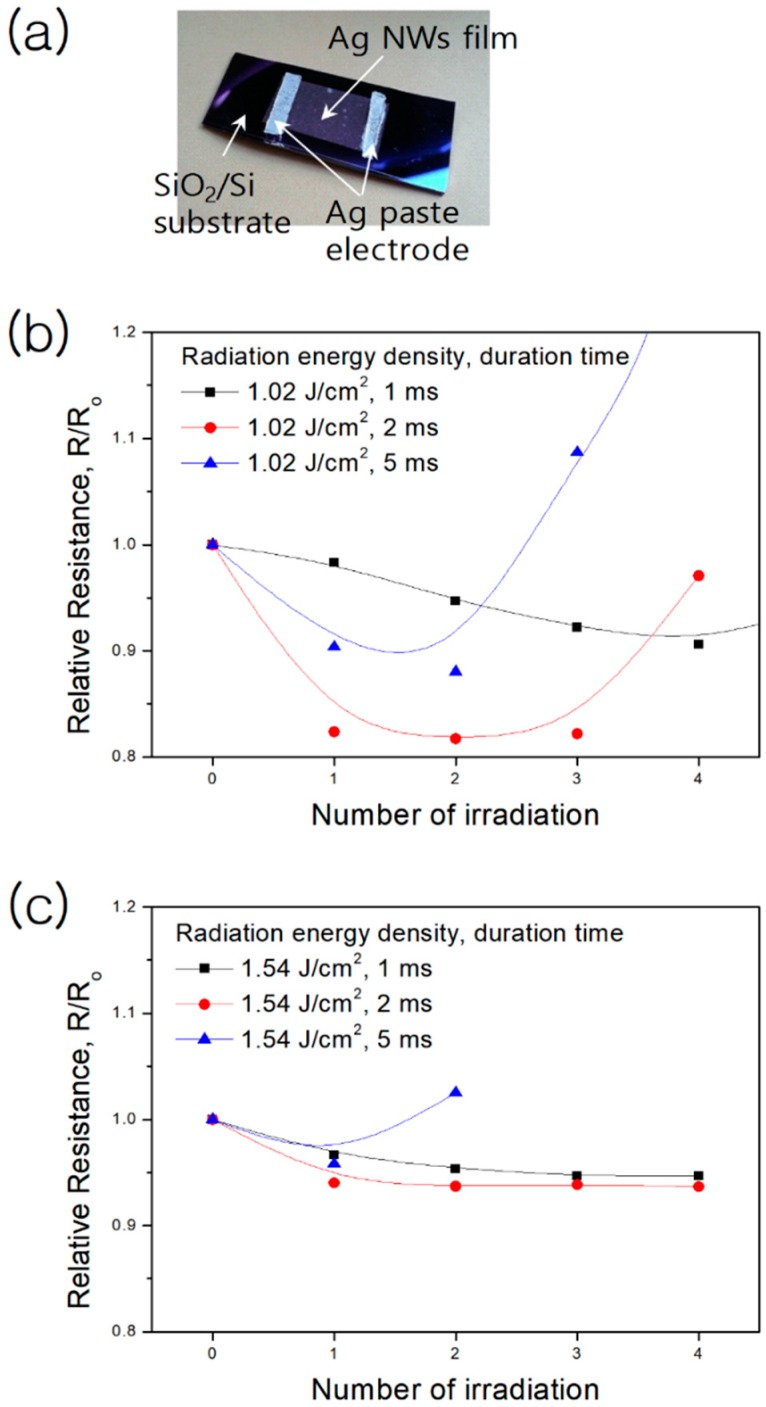
(**a**) Photograph of an Ag NW network deposited on a Si/SiO_2_ substrate. Variation of the electrical resistance of the Ag NW network during repeated flash light irradiation as a function of the number of irradiations for an energy density of (**b**) 1.02 J/cm^2^ and (**c**) 1.54 J/cm^2^ in air at room temperature.

To evaluate the electrical resistance change of the Ag NW network in terms of the flash light sintering process conditions, we deposited the Ag NW network on the Si/SiO_2_ substrate and added the Ag paste on the ends of substrate for tracking the electrical resistance change by the flash light applying conditions, as shown in Figure 3a. The samples were prepared depending on the irradiation energy, duration time, and number of irradiations. At the relatively low irradiation energy density of 1.02 J/cm^2^, a reduced electrical resistance was observed at the samples under a duration time of 2 ms (Figure 3b). At the lowest irradiation energy density, 1 ms of irradiation was not enough for the formation of welded connections between Ag NWs. Moreover, a relatively high electrical resistance was introduced even after repeated irradiation. For longer duration times, the electrical resistance was decreased by inter-connected Ag NWs, but some Ag NWs hindered electrical conductivity. It was also observed that the highly increased electrical resistance originated from further fragmentation of NWs by repeated irradiation. For a moderate duration time of 2 ms, the electrical resistance significantly decreases to ~80% of the original electrical resistance, indicating that this duration time is the optimum duration time for the formation of well-connected NWs. At the highest irradiation energy density of 1.54 J/cm^2^, the decrease in electrical resistance is limited and saturated by the combined effects of inter-connected junctions and NWs breaking (Figure 3c). For high irradiation energy densities and long duration times, the formation of isolated NWs is intensified, corresponding to an increased electrical resistance for repeated irradiation. It should be noted that the electrical conductivity of Ag NW networks can be enhanced by optimizing the irradiation energy and duration time during the flash light sintering process.

**Figure 4:**
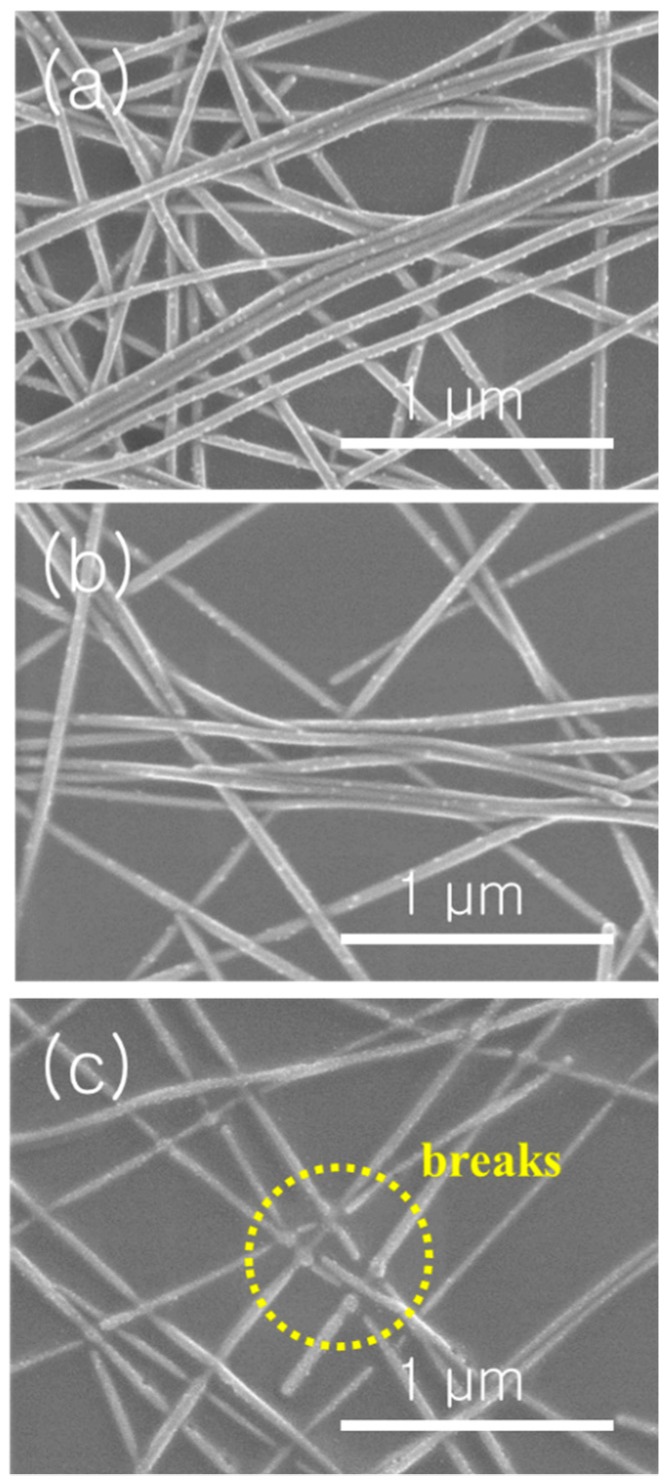
SEM images of Ag NW networks after irradiation under an irradiation energy density of 1.02 J/cm^2^ and a duration time of (**a**) 1 ms, (**b**) 2 ms, and (**c**) 5 ms.

To support the relationship between the electrical conductivity and morphological changes of the NW network during irradiation, an SEM analysis of the samples with respect to the duration time was conducted, as shown in Figure 4. At a duration time of 1 ms under an irradiation energy density of 1.02 J/cm^2^, the welding process of Ag NWs is not completed because of insufficient irradiation time. This implies that a high electrical resistance remains after repeated irradiation under the 1 ms-1.02 J/cm^2^ condition (Figure 3b). We have already confirmed from the electrical conductivity measurement that the optimum condition of flash light sintering corresponds to an irradiation energy density of 1.02 J/cm^2^ and an irradiation time of 2 ms. Under this condition, we cannot directly distinguish the difference between the SEM images of 1 ms and 2 ms (Figure 4a,b), but the morphology of welded structure will be discussed on the TEM analysis section. Under the 5 ms-1.02 J/cm^2^ condition, the Ag NWs are broken and electrical resistance is significantly increased. We conclude that the trend in morphological changes of Ag NWs as a function of the applied energy during the flash light sintering process is consistent with the trend in the electrical resistance of Ag NW networks.

**Figure 5:**
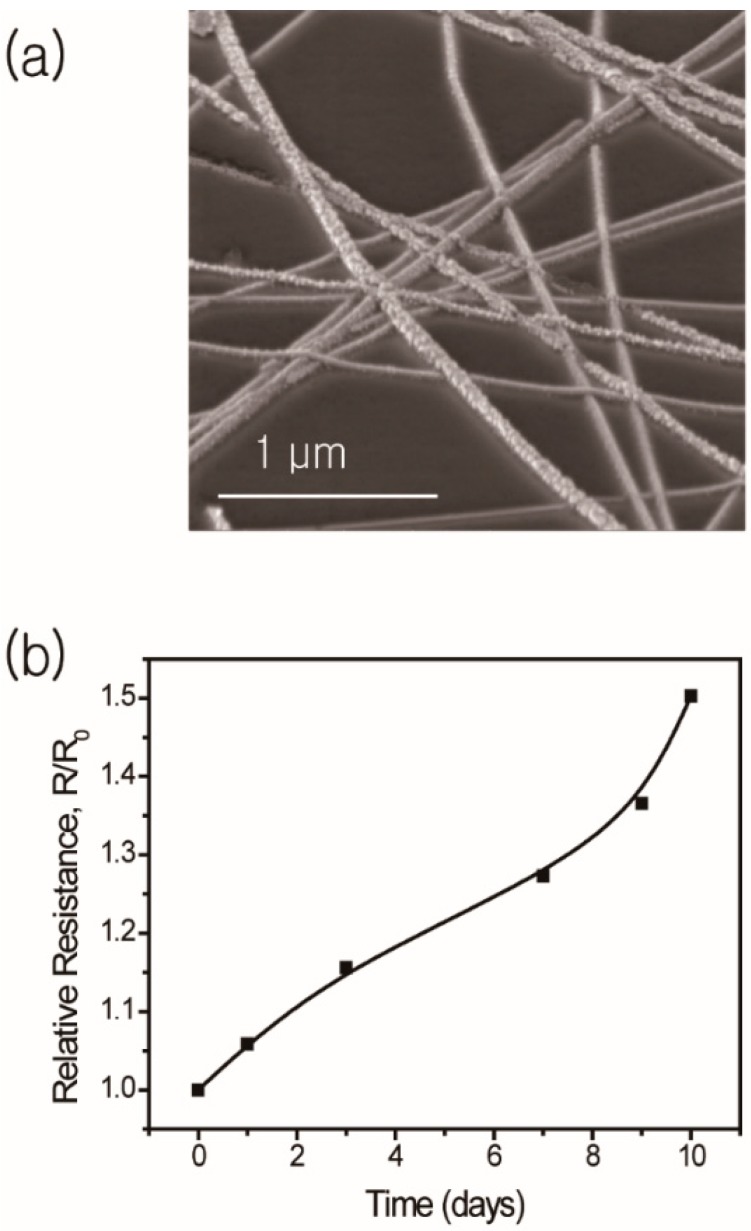
(**a**) SEM image of the Ag NW network by sulfidation and (**b**) the change in relative electrical resistance of the Ag NW network after 10-day exposure to air.

When a Ag NW network is directly exposed to ambient conditions, atmospheric sulfur such as hydrogen sulfide (H_2_S) or carbonyl sulfide (OCS) can easily react with the intrinsic defect structure of the Ag NW pentagon, which leads to a significant increase in electrical resistance. [32,33,34,35,36] This is one of the most important issues with Ag NW networks for practical applications. We also track the electrical resistance of the Ag NW network in terms of days of exposure. Figure 5a shows the SEM images after 10 days of exposure to ambient conditions. We can easily find nanoparticles that exhibit a typical morphological feature of Ag NW sulfidation. Compared to other reports [37,38], our flash light-sintered Ag NWs show a relatively low increase in relative resistance without any protection layer (see Figure 5b), which is caused by the removal of the strain on the junction of the Ag NW network. The 1.5-fold increase in electrical resistance could also give a significant problem for expanding the applications, which should be considered on other researches.

**Figure 6:**
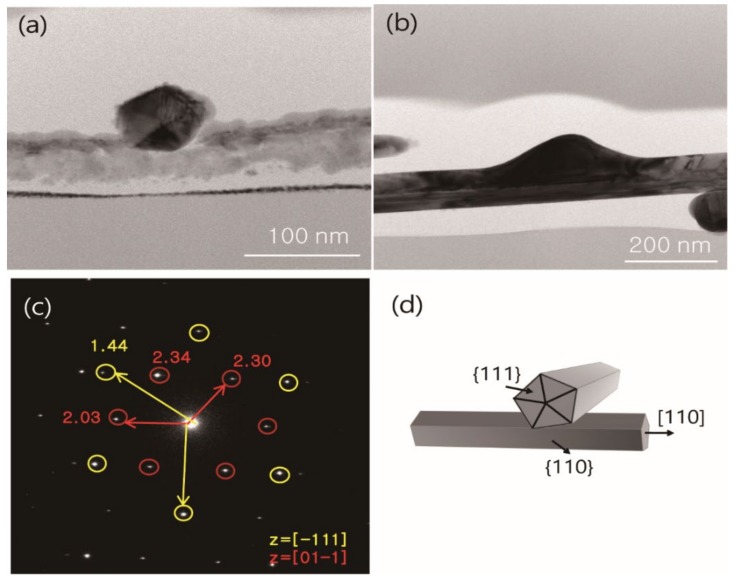
TEM images of Ag NWs (**a**) before and (**b**) after the flash light sintering process; (**c**) selected area electron diffraction (SAED) pattern of the junction of welded Ag NWs; (**d**) schematic diagram of two welded nanowires.

A TEM analysis has been conducted for obtaining the junction structure before/after the flash light welding process. Figure 6a shows the two inter-connected Ag NWs after the coating process. Each Ag NW remains an individual structure, and the pentagonal structure of [111] direction of the Ag NWs is clearly observed [39]. Under the optimum condition (1.02 J/cm^2^, 2 ms) of the flash light sintering process, the junction structure is completely changed to the mixed-phase structure of two Ag NWs. The selected area electron diffraction (SAED) pattern of the welded Ag NWs has two different indices, i.e., [111] and [110], which correspond to the parallel and perpendicular direction of Ag NWs growth (Figure 6c). While the upper Ag NW morphology assumes an amorphous structure, the structure remains identical to the individual Ag NWs. Figure 6d shows a schematic diagram of the direction of the welded Ag NWs that supports crossing and networking. The optimal flash sintering is suitable for fabricating Ag NW networks that have low contact and electrical resistance.

## 4. Conclusions

In summary, we performed the flash light sintering process to investigate the welding mechanism of Ag NW networks to effectively reduce the electrical resistance. The optimised condition of the flash light sintering process could reduce the electrical resistance by ~20%, more stable than other papers [37,38] without any protection layer. After flash light sintering, the junction structure of the Ag NWs changed to the welded structure which was identical to the original Ag NW crystal structures significantly reducing the electrical resistance. Furthermore, with this process, a wielded structure with low electrical resistance can be obtained rapidly. Therefore, this technique could be applied to the mass production of Ag NW networks for transparent electrode areas.
